# IL-9-triggered lncRNA Gm13568 regulates Notch1 in astrocytes through interaction with CBP/P300: contribute to the pathogenesis of experimental autoimmune encephalomyelitis

**DOI:** 10.1186/s12974-021-02156-5

**Published:** 2021-05-11

**Authors:** Xiaomei Liu, Feng Zhou, Weixiao Wang, Guofang Chen, Qingxiu Zhang, Ruixue Lv, Zijun Zhao, Xiangyang Li, Qian Yu, Jessica M. Meves, Hui Hua, Xiaocui Li, Xiaotian Wang, Hong Sun, Dianshuai Gao

**Affiliations:** 1 Jiangsu Key Laboratory of Immunity and Metabolism, Department of Pathogen Biology and Immunology and Laboratory of Infection and Immunity, Xuzhou Medical University, 209 Tongshan Road, Xuzhou, Jiangsu 221004 People’s Republic of China; 2 Neurology Department, The Affiliated Xuzhou Center Hospital of Nanjing University of Chinese Medicine, Xuzhou, People’s Republic of China; 3 Neurology Department, Xuzhou Central Hospital, Xuzhou, People’s Republic of China; 4 Neurology Department, Xuzhou Clinical School of Xuzhou Medical University, Xuzhou, Jiangsu 221009 People’s Republic of China; 5 Department of Neurology, Second Affiliated Hospital of Xuzhou Medical University, Xuzhou, Jiangsu 221006 People’s Republic of China; 6 Department of Psychiatry, University of Michigan Medicine, MI48109, Ann Arbor, Michigan USA; 7 Department of Physiology, Xuzhou Medical University, Xuzhou, Jiangsu 221004 People’s Republic of China; 8 Xuzhou Key Laboratory of Neurobiology, Department of Neurobiology and Anatomy, Xuzhou Medical University, Xuzhou, Jiangsu 221004 People’s Republic of China

**Keywords:** IL-9, Astrocytes, LncRNA Gm13568, Notch1, Inflammatory cytokines, Experimental autoimmune encephalomyelitis (EAE)

## Abstract

**Background:**

Interleukin 9 (IL-9), produced mainly by T helper 9 (Th9) cells, has been recognized as an important regulator in multiple sclerosis (MS) and its animal model, experimental autoimmune encephalomyelitis (EAE). Astrocytes respond to IL-9 and reactive astrocytes always associate with blood-brain barrier damage, immune cell infiltration, and spinal injury in MS and EAE. Several long non-coding RNAs (lncRNAs) with aberrant expression have been identified in the pathogenesis of MS. Here, we examined the effects of lncRNA Gm13568 (a co-upregulated lncRNA both in EAE mice and in mouse primary astrocytes activated by IL-9) on the activation of astrocytes and the process of EAE.

**Methods:**

In vitro, shRNA-recombinant lentivirus with glial fibrillary acidic protein (GFAP) promoter were performed to determine the relative gene expression and proinflammatory cytokines production in IL-9 treated-astrocytes using Western blot, real-time PCR, and Cytometric Bead Array, respectively. RIP and ChIP assays were analyzed for the mechanism of lncRNA Gm13568 regulating gene expression. Immunofluorescence assays was performed to measure the protein expression in astrocytes. In vivo, H&E staining and LFB staining were applied to detect the inflammatory cells infiltrations and the medullary sheath damage in spinal cords of EAE mice infected by the recombinant lentivirus. Results were analyzed by one-way ANOVA or Student’s *t* test, as appropriate.

**Results:**

Knockdown of the endogenous lncRNA Gm13568 remarkably inhibits the Notch1 expression, astrocytosis, and the phosphorylation of signal transducer and activator of transcription 3 (p-STAT3) as well as the production of inflammatory cytokines and chemokines (IL-6, TNF-α, IP-10) in IL-9-activated astrocytes, in which Gm13568 associates with the transcriptional co-activators CBP/P300 which are enriched in the promoter of Notch1 genes. More importantly, inhibiting Gm13568 with lentiviral vector in astrocytes ameliorates significantly inflammation and demyelination in EAE mice, therefore delaying the EAE process.

**Conclusions:**

These findings uncover that Gm13568 regulates the production of inflammatory cytokines in active astrocytes and affects the pathogenesis of EAE through the Notch1/STAT3 pathway. LncRNA Gm13568 may be a promising target for treating MS and demyelinating diseases.

**Supplementary Information:**

The online version contains supplementary material available at 10.1186/s12974-021-02156-5.

## Background

Multiple sclerosis (MS) is a chronic inflammatory autoimmune disease of the central nervous system (CNS), in which immune cells infiltrate into the white matter associated with demyelination and axonal loss [1]. The occupancy of T cells and glia cells in patient lesions suggests that both are important contributors to MS [2, 3]. Autoreactive effector CD4^+^T cells, including proinflammatory Th1 cell and Th17 cell, are believed to be the major effector cells [4], which migrate into the CNS and initiate the MS process. Recently, Th9 cell and its secreting cytokine IL-9 have been implicated in MS, which activates and cooperates with other CD4^+^T cells and CNS-resident cells in MS and experimental autoimmune encephalomyelitis (EAE), an animal model of MS [5].

Astrocytes, the most abundant cell population in the CNS, play an essential role in the homeostasis of CNS. Astrocytes participate in the formation and integrity of the blood-brain barrier (BBB), support of neurons, and regulation of axonal outgrowth. When CNS injury occurs, astrocytes become activated, which are clearly identified by the dramatically upregulated expression of glial fibrillary acidic protein (GFAP) [6, 7]. In response to IL-9 stimulation, astrocytes aggravate the BBB disruption, increase chemokine production and facilitate T cell migration into the CNS [8, 9]. However, the mechanisms by which astrocyte activation with IL-9 stimulation contribute to MS remains undefined.

The Notch signaling is a fundamental and well-conserved pathway associated with cell fate. The canonical Notch pathway arises from the interaction of transmembrane Notch receptors (Notch1- 4) with ligands (Jagged1, Jagged2, DLL1, DLL3 or DLL4), whereas the non-canonical pathway is activated through various molecules, such as NF-κB, STAT3 [10, 11]. Upon ligand binding or activation, Notch1 is cleaved and releases Notch1 intracellular domain (NICD) that directly translocates into the nucleus to form a complex with RBP/J and mastermind like (MAML) proteins, which promotes the transcription of target genes [10, 11]. In addition, NICD binds to the serine residue of phosphorylated STAT3 (p-STAT3), facilitates the nuclear translocation of p-STAT3, and initiates the transcription of corresponding target genes [12, 13]. It has been reported that Notch is associated with several progressive neurodegenerative diseases, including MS, amyotrophic lateral sclerosis (ALS) and Alzheimer's disease (AD). Notch1 signaling activation increases astrocyte proliferation and enhances reactive astrogliosis after CNS injury [14, 15].

Long noncoding RNAs (lncRNAs), ranging from hundreds to tens of thousands of nucleotides in length with no protein-coding capacity, have been proved to play vital roles in diverse biological processes of genomic imprinting and development to immune response [16]. LncRNAs are involved in neurodegenerative diseases, including MS, ALS, AD, Parkinson’s disease (PD), and Huntington’s disease (HD) [17, 18]. Importantly, differentially expressed-lncRNAs in activated astrocytes are closely related to EAE progression [19]. However, the mechanisms underlying how lncRNAs regulate astrocytes activation and MS process are still poorly understood.

The transcriptional co-activators CREB-binding protein (CBP) and P300 are histone acetyltransferase (HAT) contributing to histone 3 lysine 27 acetylation (H3K27ac), RNA Pol II acetylation, and NF-κB enrichment in the promoter region of target genes, which forms a transcription network to facilitate the expression of target gene [20–24]. Lately, it has been reported that CBP/P300 binds directly to RNA to enhance histone acetylation and gene transcription [25]. Additionally, lncRNAs are associated with the CBP/p300 complex and RNA polymerase II, which regulate gene expression [24, 26].

In previous study, we analyzed lncRNA expression profiles in EAE mice as well as in activated astrocytes with IL-9 stimulation [19]. Here, we aim to further explore the function and mechanism of lncRNA Gm13568 (a co-upregulated lncRNA in EAE mice and IL-9-activated astrocytes) in regulating astrocytes activation and EAE progression. We display that Gm13568 contributes to the production of inflammatory cytokines and chemokines in activated astrocytes through interaction with CBP/P300, which is enriched in the promoter of Notch1 and promotes Notch1 expression and signaling activation, thereby aggravating the EAE damage. These results suggest that targeting lncRNAs in astrocytes is an attractive option for the treatment of MS.

## Methods

### Animal, antibodies, and reagents

C57BL/6 mice were purchased from the Shanghai Experimental Animal Center, Chinese Academy of Sciences. All experimental procedures described in our study were carried out based on the Provision and General Recommendation of the Chinese Laboratory Association. The protocol was approved by the Laboratory Animal Ethics Committee of Xuzhou Medical University (201801w005). All mice we bred under specific pathogen-free conditions. Antibodies used in this study were as follows: anti-IL-9 antibody (Rabbit, ab203386, Abcam), anti-GFAP antibody (Rabbit, ab7260, Abcam), anti-GFAP (Mouse, ab4648, Abcam), anti-cleaved Notch1 antibody (NICD, Rabbit, 4147, Cell Signaling Technology), anti-Notch 1 antibody (Goat, sc-6014, SANTA CRUZ), anti-p-STAT3 (Rabbit, Tyr705, BS4181, Bioworld Technology), anti-STAT3 (Rabbit, AP0365, Bioworld Technology), NF-κB p65 (Rabbit, 8242, Cell Signaling Technology), anti- CBP/P300 antibody (Rabbit, 4771s, Cell Signaling Technology), anti-RNA Pol II antibody (Mouse, 17-620, Sigma Aldrich), and anti-H3K27ac antibody (Rabbit, ab177178, Abcam). The secondary antibodies were all purchased from Sigma Biotechnology. Myelin oligodendrocyte glycoprotein (MOG) amino acids 35-55 (MOG_35-55_ peptides, MEVGWYRSPFSRVVHLYRNGK) were purchased from China Peptides Co. Ltd (shanghai, China). Recombinant mouse IL-9 was from R&D Systems; multiplex magnetic bead-based antibody detection kits (cytokine and chemokine detection kits, Cytometric Bead Array) were from BD Biosciences.

### EAE induction and clinical evaluation

C57BL/6 female mice (6–8 weeks old) were immunized with 250 μg MOG_35-55_ emulsified in complete Freund’s adjuvant (Sigma Aldrich) containing 5 mg/ml heat-killed Mycobacterium tuberculosis (H37Ra strain, Difco). On day 0 and day 2, mice were intraperitoneally received pertussis toxin (200 ng, Invitrogen), and then mice were monitored daily for clinical signs of EAE according to the following grading scale in a double-blinded manner: 0, no clinical signs; 1, limp tail; 2, paraparesis (weakness, incomplete paralysis of one or two hind limbs); 3, paraplegia (two hind limbs paralysis); 4, paraplegia with fore limb weakness or paralysis; and 5, moribund or death. The PBS with the same volume was injected into mice as the negative control.

### Primary astrocyte culture

Primary mouse astrocytes were separated and cultured as described previously [27]. Astrocytes were synchronized with non-serum culture media for 12 h before IL-9 stimulation.

### Real-time PCR assay

Total RNA from the spinal cords of mouse or astrocytes was extracted by Trizol reagent (Invitrogen). Real-time PCR assay was conducted as described previously [28]. The relative genes transcription was calculated with the 2^−ΔΔCT^ method. The primers are listed in Supplementary Table S1.

### Cytometric Bead Array (CBA) assay

The serum of mice or the supernatant of cultured astrocytes was collected. The secretion levels of IL-6, TNF-α, interferon-inducible protein-10 (IP-10), and monocyte chemoattractant protein-1 (MCP-1) were detected by CBA assay (BD Biosciences, USA) as described previously [29].

### Western blot assay

As described previously [27], the total protein was extracted from the spinal cords of mouse as well as the cultured astrocytes. The expression level of protein in samples was normalized by β-actin protein.

### Lentivirus generation

Recombinant lentiviral vectors carrying EGFP and astrocyte-specific promoter of glial fibrillary acidic protein (GFAP) were constructed by Gene Chem (Shanghai, China) and were verified (Supplementary Fig. S1). Because the sequence of Gm13568 completely complements the sense chain at the 9075-9497 locus of the Notch1 gene, conventional shRNA knockdown may simultaneously affect the expression of endogenous Notch1 gene. We constructed recombinant lentivirus vectors with GFAP promoter that overexpresses the 9075-9497 sequence of Notch1 gene to make them competitively bind to Gm13568 (LV-Inhibit-Gm13568), so as to inhibit the effect of endogenous Gm13568 on Notch1 expression.

### RNA Immunoprecipitation (RIP) assay

RIP assay was performed as previously described [30]. In brief, after treating with IL-9, 2 × 10^7^ primary mouse astrocytes were treated with 0.3% formaldehyde in culture medium, and then 0.125 M Glycine was added to the medium. Cells were washed in cold PBS and pelleted. The pellets were resuspended in RIPA buffer and incubated on ice, and the lysate was centrifugated at 13,000 RPM for 10 min. Antibodies mixing Protein A/G beads were added and incubated at 4 °C overnight. Next, the beads were resuspended in RIPA buffer and were treated with proteinase K at 45 °C for 45 min. Finally, samples were extracted with Trizol and then RNA was detected by real-time PCR assay. The data of retrieved RNAs were calculated by the subtraction of RT/input ratio and non-RT/input ratio. The primer sequence of Gm13568 was listed in Supplementary Table S1.

### Chromatin immunoprecipitation (ChIP) assay

ChIP assays were performed as described previously [30]. Primary mouse astrocytes were treated with IL-9 for 6 h. 1 × 10^7^ cells were used for each ChIP-enrichment. Chromatin was sheared to the fragment size of 200–500 bp. The antibodies were anti-NF-κB p65, anti-H3K27ac, anti-RNA Pol II, anti-CBP/P300, and normal mouse IgG. All immunoprecipitated chromatin DNA was analyzed by real-time PCR. The primer sequences were listed in Supplementary Table S1.

### Histopathology assay

Histopathology assay was performed as previously described [27, 31]. Hematoxylin and eosin (H&E) or luxol fast blue (LFB) staining on 4 μm paraffin-embedded spinal cord sections were used in evaluating inflammation and demyelination, respectively.

### Statistical analysis

Statistical analysis was performed by GraphPad Prism version 5.0 software with the two-tailed unpaired Student's *t* test or one-way multiple-range analysis of variance (ANOVA). A Mann-Whitney test was used for nonparametric data (EAE scoring). Data presented as mean ± standard error of mean (SEM), calculated for all points from at least three independent experiments in triplicates. Values of *p* < 0.05 was considered significant.

## Results

### The production of IL-9 and inflammatory cytokines as well as the expression of NICD and p-STAT3 are increased in EAE mice

**Fig. 1:**
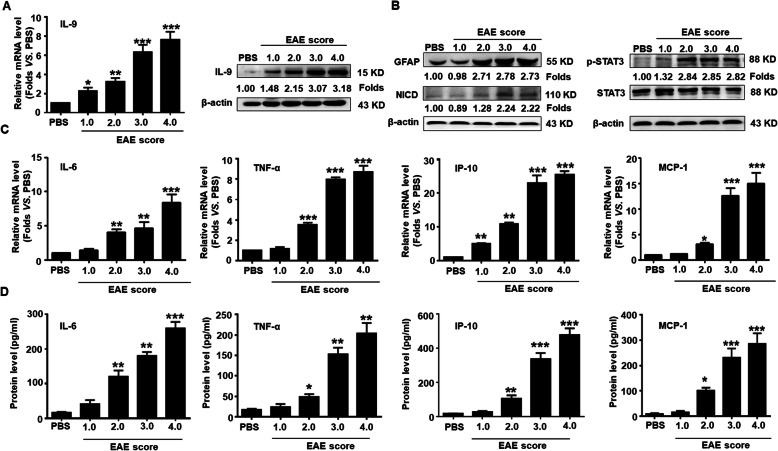
Upregulation of IL-9 and inflammatory cytokines as well as the activation of Notch1 signaling during EAE process. **a** The level of IL-9 in spinal cords of EAE mice with different clinical scores was measured by real-time PCR and Western blot assay, respectively. **b** The expressions of GFAP, NICD and p-STAT3 in spinal cords along with EAE process were detected by Western blot assay. **c** The mRNA levels of IL-6, TNF-α, IP-10, and MCP-1 in spinal cords were determined using real-time PCR. **d** The secretion levels of IL-6, TNF-α, IP-10 and MCP-1 in the sera were detected by Cytometric Bead Array (CBA) during the EAE process. *p < 0.05, **p < 0.01 and ***p < 0.001 versus PBS group (*n* = 6/group, one-way ANOVA). These results were repeated four times. Data are represented as the mean ± SEM

It has been recently demonstrated that Th9/IL-9 is a key player in regulating autoimmune responses in MS and EAE accompanied by the production of a large number of inflammatory cytokines. To investigate the potential role of IL-9 in inducing Notch1 pathway activation, reactive astrogliosis, and the production of inflammatory cytokines in EAE mice, we firstly measured the expression of IL-9 in the tissues of spinal cords. As shown in Fig. 1a, the mRNA and protein level of IL-9 was gradually increased in the spinal cords along with the severity of EAE. Then, the expression of GFAP, NICD, and p-STAT3 was detected by Western blot assay. The results showed that the expression of GFAP protein was markedly elevated in the spinal cords of EAE mice with score 2.0 and lasted until score 4.0. Similarly, the expression of NICD and the phosphorylation level of STAT3 (p-STAT3) was also significantly upregulated from EAE score 2.0 to score 4.0 (Fig. 1b). Finally, the mRNA transcription and secretion of pro-inflammatory cytokines and chemokines (IL-6, TNF-α, IP-10, and MCP-1) in the spinal cords and peripheral blood of EAE mice were assessed by real-time PCR and CBA assay, respectively (Fig. 1c, d). The production of IL-6, TNF-α, IP-10, and MCP-1 was obviously increased along with EAE process, which was consistent with our previous study [27]. These results suggest whether IL-9 secreted by Th cells stimulates astrogliosis and Notch1 signal activation, therefore promoting the production of pro-inflammatory cytokines and chemokines in the CNS of EAE mice.

### IL-9 stimulates astrocytes to promote Notch1 pathway activation and inflammatory cytokine production

**Fig. 2:**
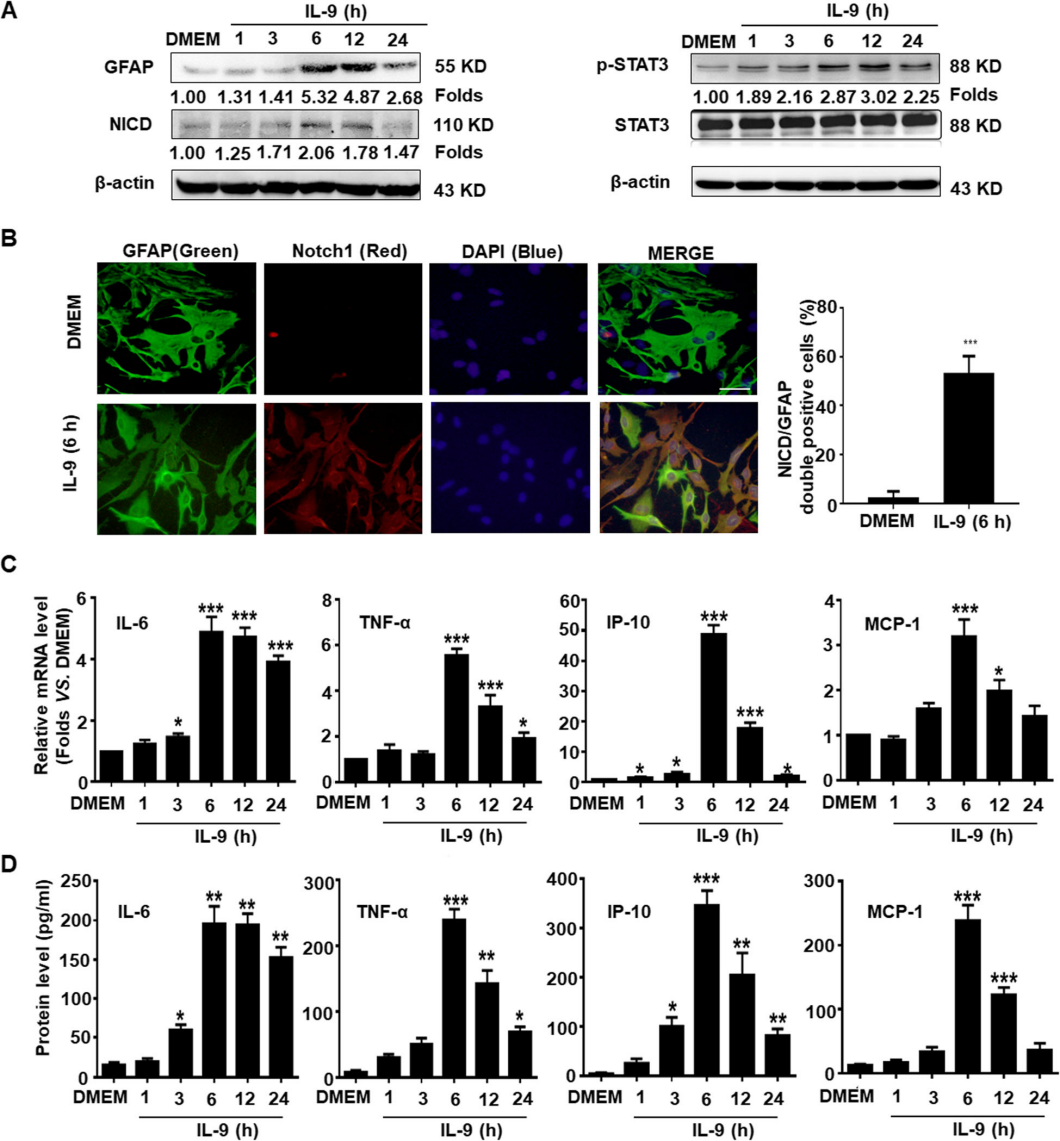
IL-9 activates the Notch1 pathway and promotes inflammatory cytokines production in astrocytes. Primary mouse astrocytes were incubated in a serum-free medium overnight followed by treating with IL-9 at the indicated time point. **a** The expression changes of GFAP, NICD, and p-STAT3 were analyzed by Western blot assay. **b** Immunofluorescent staining for GFAP (green), Notch1 (red), and nuclear staining of DAPI (blue) in cultured astrocytes with IL-9 treatment for 6 h. Scale bars, 50 μm. **c** The mRNA levels of IL-6, TNF-α, IP-10, and MCP-1 in astrocytes were detected using real-time PCR assay. **d** The secretion levels of IL-6, TNF-α, IP-10, and MCP-1 in the supernatant of astrocytes were measured by CBA assay. *p < 0.05, **p < 0.01 and ***p < 0.001 versus DMEM group (one-way ANOVA). The data are from three independent experiments and represented as the mean ± SEM

To explore the role of IL-9 in Notch1 pathway activation and inflammatory cytokine production in astrocytes, primary mouse astrocytes were treated with IL-9 in serum-free media. Western blot results showed that the expression of GFAP was dramatically increased at 6 h and lasted for 24 h in the astrocytes with IL-9 treatment. Meanwhile, the expressions of NICD and p-STAT3 were elevated at 6 h after IL-9 stimulation (Fig. 2a). The results of an immunofluorescence assay indicated that the expression of Notch1 in cytoplasm was also significantly increased in IL-9 stimulated-astrocytes, compared to the DMEM group (Fig. 2b). In addition, the mRNA transcription and secretion levels of inflammatory cytokines such as IL-6, TNF-α, IP-10, and MCP-1 were simultaneously upregulated and peaked at 6 h in the astrocytes stimulated by IL-9 (Fig. 2c, d). These data suggest that IL-9 activates the Notch1/ STAT3 signaling pathway and promotes the productions of pro-inflammatory cytokines in astrocytes.

### Knockdown of Notch1 suppresses inflammatory response in astrocytes by IL-9

**Fig. 3:**
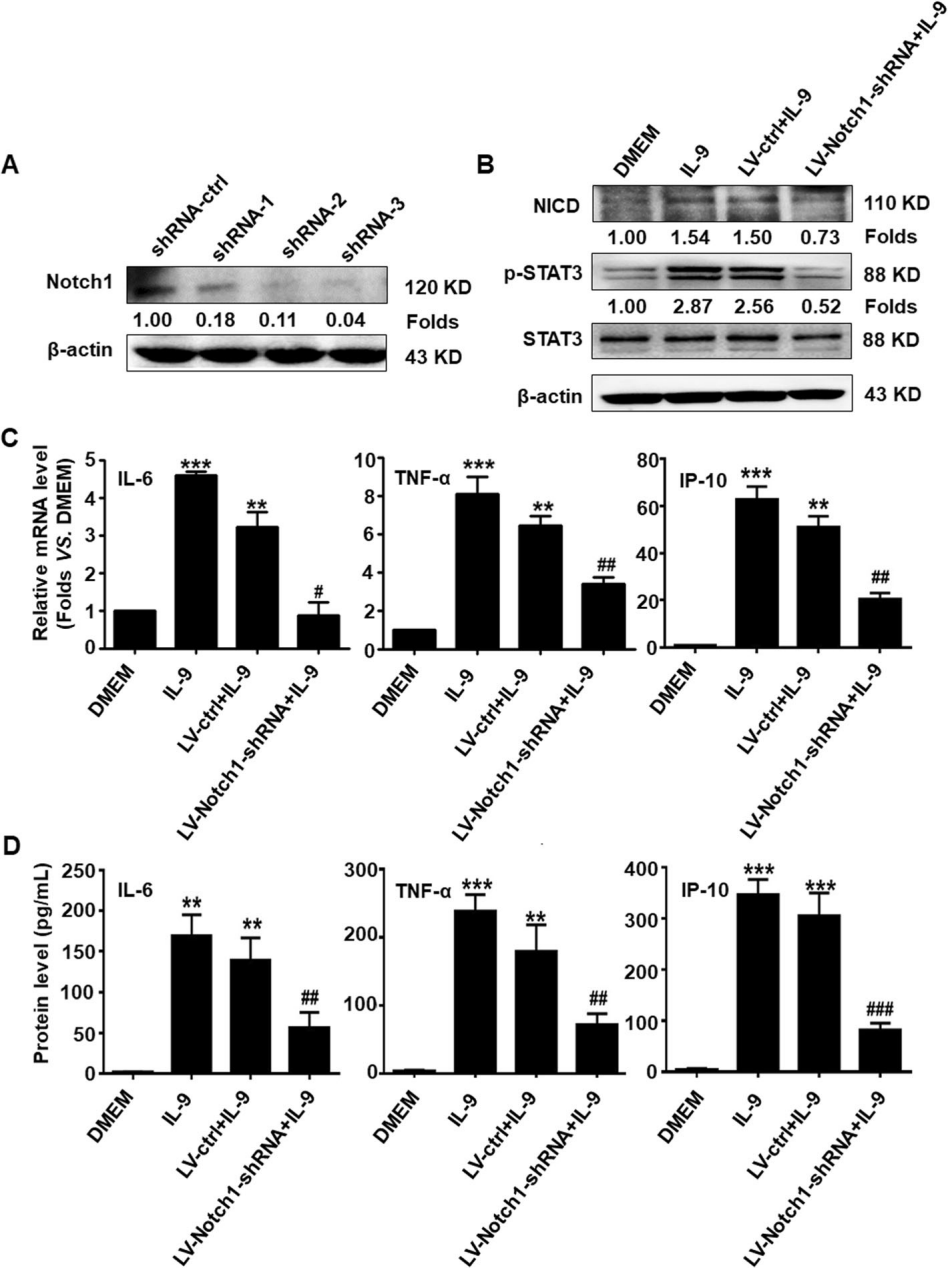
Knockdown of Notch1 reduces inflammatory cytokine production in activated-astrocytes by IL-9. **a** The 3 recombinant lentivirus plasmids of Notch1-shRNA targeting astrocytes were transduced into mouse primary astrocytes using the Neon™ electron transfection system (MPK5000) for 48 h. The expression of Notch1 protein in astrocytes was evaluated by Western blot assay. **b** Primary mouse astrocytes were infected with recombinant lentivirus (LV-Notch1-shRNA, LV-ctrl) for 72 h, and then incubated in a serum-free medium overnight followed by treating with IL-9 for 6 h. Western blot analysis for the proteins of Notch1 pathway. **c** The mRNA levels of IL-6, TNF-α, and IP-10 in astrocytes were measured by real-time PCR. **d** The secretion levels of IL-6, TNF-α, and IP-10 in the supernatant of astrocytes were detected by CBA assay. **p < 0.01 and ***p < 0.001 versus DMEM group; ^#^*p* < 0.05 and ^##^*p* < 0.01 and ^###^*p* < 0.001 versus LV-ctrl+IL-9 group (one-way ANOVA, the two-tailed Student’s *t* test). The data are from three independent experiments and represented as mean ± SEM

To further investigate whether Notch1 is involved in regulating inflammatory responses in IL-9 stimulated-astrocytes, recombinant lentiviral vectors of three Notch1-shRNA (LV-Notch1-shRNA) specifically targeting astrocytes were constructed and transfected into primary mouse astrocytes, respectively. As shown in Fig. 3a, the Notch1 protein level was significantly repressed in Notch1-shRNA-1, shRNA-2, and shRNA-3 groups, particularly in the shRNA-3 group, compared with the shRNA-ctrl group. Thus, the recombinant lentiviral vector of Notch1-shRNA-3 was selected for packaging and infection. Western blot results showed that the expression of NICD and the phosphorylation level of STAT3 were obviously decreased from LV-Notch1-shRNA infection group in IL-9 treated-astrocytes, compared to the LV-ctrl group (Fig. 3b). Furthermore, the production of inflammatory cytokines including IL-6, TNF-α, and IP-10 was markedly reduced in LV-Notch1-shRNA infected-astrocytes (Fig. 3c, d). These results indicate that IL-9-induced upregulation and activation of Notch1 contribute to the inflammatory cytokines production in astrocytes.

### Identification of lncRNA Gm13568 targeting Notch1

**Fig. 4:**
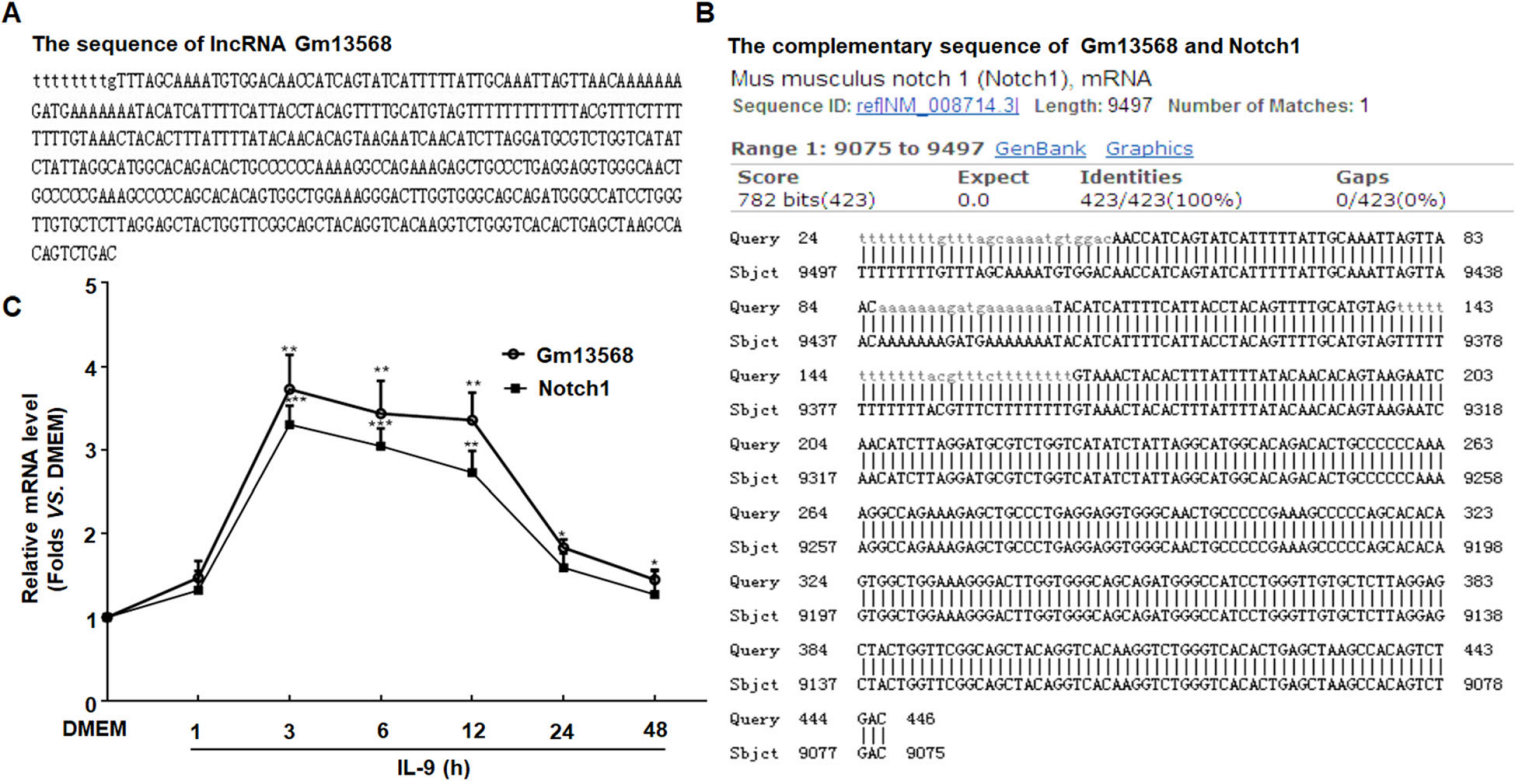
Identification of lncRNA targeting the Notch1 gene. **a** The sequence of lncRNA Gm13568 with 446 base length. **b** The sequence of Gm13568 is complementary to the sense chain of Notch1 gene at 9075-9497 locus. **c** Primary mouse astrocytes were treated in a serum-free medium overnight followed by stimulating with IL-9 at different time period. Real-time PCR was performed to detect the change levels of Gm13568 and Notch1 in astrocytes. *p < 0.05 and **p < 0.01 and ***p < 0.001 versus DMEM (one-way ANOVA). Results are represented as mean ± SEM

In our previous study, the changes of lncRNA profiles were reported both in EAE mice (in vivo) and in activated astrocytes with IL-9 stimulation (in vitro) [19]. We further found that the sequence of ENSMUST00000156099 (Gm13568, one of the lncRNA co-upregulated in vivo and in vitro) with 446 bp length is identical to the antisense sequence of Notch1 gene at the 9075-9497 locus that is complementary to the sense chain of the Notch1 gene (located between exon 2 and exon 3) (Fig. 4a, b). The time course data showed that the changes of mRNA level between Gm13568 and Notch1 mirrored one another. At 3 h of IL-9 treatment, the levels of Gm13568 and Notch1 were significantly increased and lasted for 12 h (Fig. 4c). Taken together, these results suggest that Gm13568 may have a positive regulatory effect on Notch1 expression.

### LncRNA Gm13568 regulates the Notch1 pathway and inflammatory cytokine production in activated astrocytes by IL-9

**Fig. 5:**
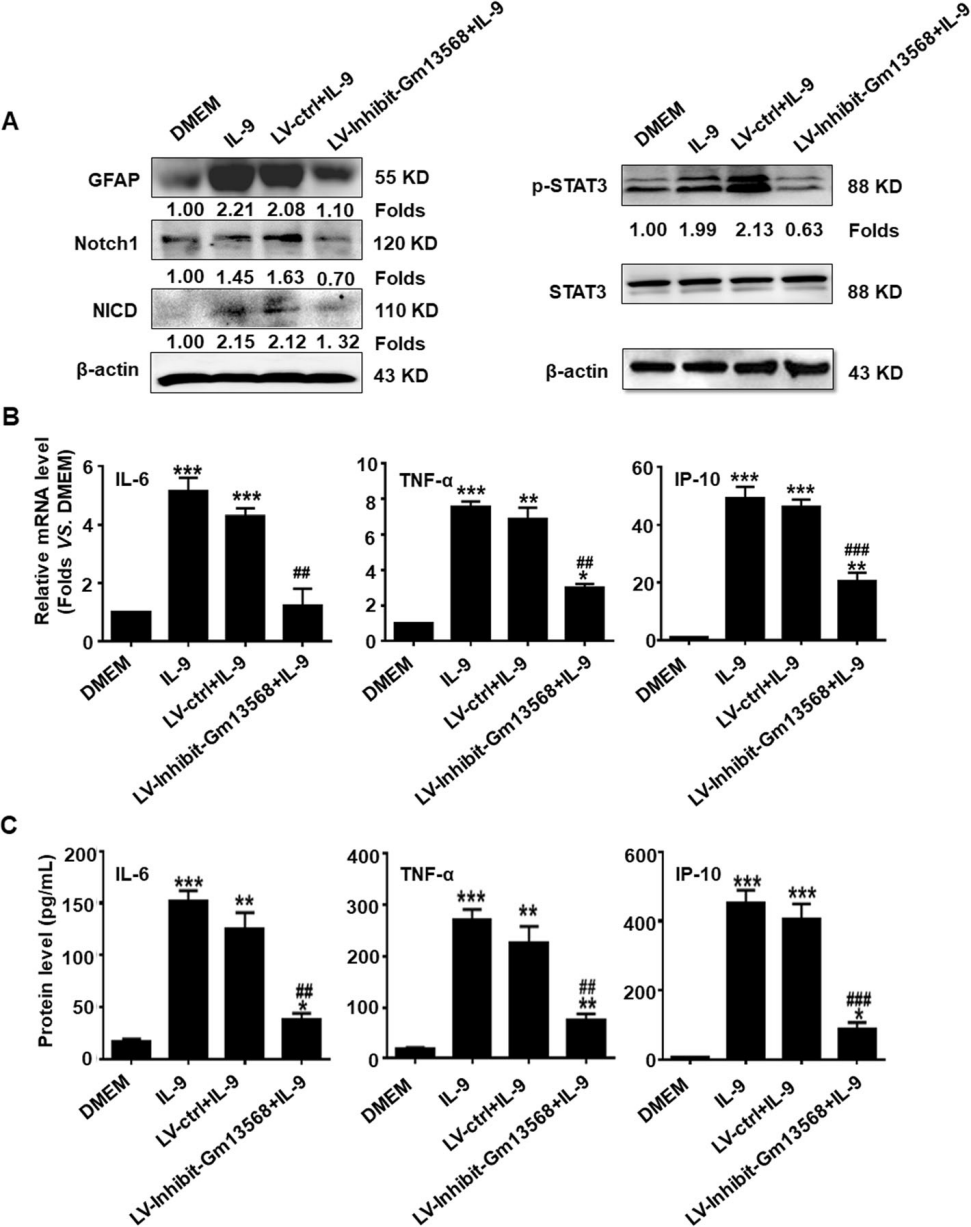
Inhibition of Gm13568 downregulates Notch1 signaling activation as well as inflammatory cytokine production in astrocytes by IL-9. **a** Primary mouse astrocytes were infected with recombinant lentivirus, LV-Inhibit-Gm13568 and LV-ctrl, for 72 h, respectively. Then, the astrocytes were incubated in a serum-free medium overnight followed by IL-9 stimulation for 6 h. Western blot assay was used for measuring the protein expressions of GFAP, Notch1/NICD, and p-STAT3. **b** The mRNA levels of IL-6, TNF-α and IP-10 in astrocytes were measured by real-time PCR assay. **c** The secretion of IL-6, TNF-α, and IP-10 in the supernatant of astrocytes were analyzed by CBA assay. The data are represented as the mean ± SEM. *p < 0.05, **p < 0.01, and ***p < 0.001 versus DMEM group; ^##^*p* < 0.01 and ^###^*p* < 0.001 versus LV-ctrl+IL-9 group (one-way ANOVA, the two-tailed Student’s *t* test)

To evaluate the role of Gm13568 in regulating Notch1 expression and signal activation and inflammatory cytokines production in activated astrocytes, we infected primary mouse astrocytes with recombinant lentivirus inhibiting Gm13568 (LV-Inhibit Gm13568) that we had constructed and packaged. In the LV-Inhibit-Gm13568 group, the expressions of GFAP and Notch1were lower after IL-9 stimulation for 6 h than that of LV-ctrl group, indicating the inhibition of Gm13568 significantly decreased the Notch1 expression and reactive astrocytosis (Fig. 5a). Similarly, the expression of NICD and p-STAT3 were also markedly decreased in LV-Inhibit-Gm13568 group (Fig. 5a). Additionally, the production of IL-6, TNF-α and IP-10 was in much lower level in the LV-Inhibit-Gm13568 group (Fig. 5b, c). These observations indicate that Gm13568 regulates the expression and signal activation of Notch1, therefore mediating the production of inflammatory cytokines in astrocytes.

### The interaction of Gm13568 with CBP/P300 regulates the Notch1 expression in astrocytes by IL-9

**Fig. 6:**
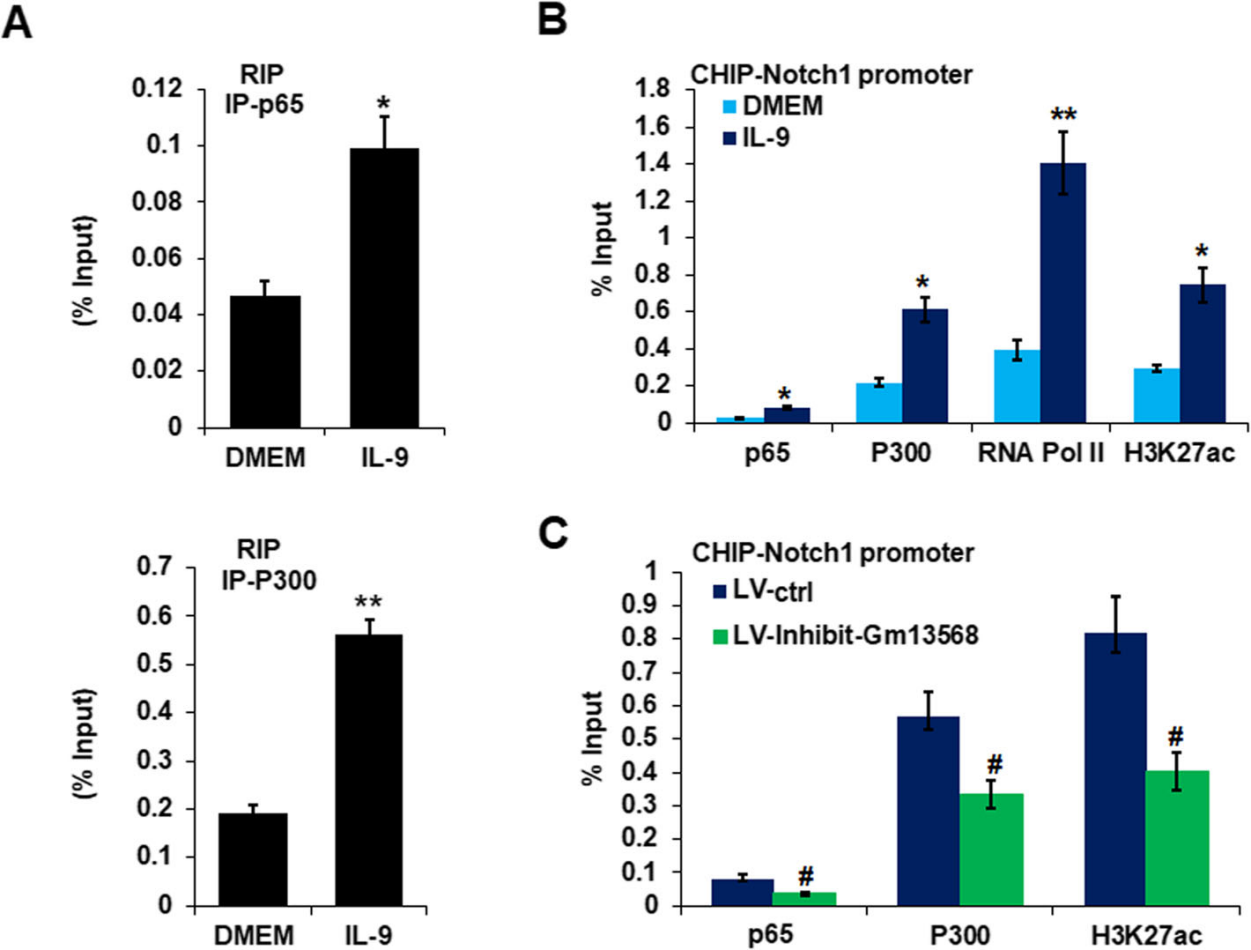
LncRNA Gm13568 interacts with CBP/P300 to regulate the expression of Notch1 in astrocytes. **a** RIP assay was performed for the interaction of Gm13568 with NF-κB p65 and CBP/P300 in astrocytes treatment with IL-9 for 6 h. The data were from three independent experiments. **b** ChIP assay analysis of NF-κB p65, CBP/P300, RNA Pol II, and H3K27ac enrichment on the promoter of Notch1 gene in astrocytes stimulation with IL-9 for 6 h. *p < 0.05, **p < 0.01, ***p < 0.001 vs DMEM (one-way ANOVA). The data are from three independent experiments. Error bars represent mean ± SEM. **c** ChIP assay analysis of NF-κB p65, CBP/P300, and H3K27ac enrichment on the promoter of Notch1 gene in astrocytes infected by LV-Inhibit-Gm13568 for 72 h, followed by stimulation with IL-9 for 6 h. ^#^*p* < 0.05 versus LV-ctrl. The data were from three independent experiments

To further explore the potential regulating mechanism of Gm13568 on Notch1 expression, the RIP assay was performed to analyze Gm13568 and NF-κB p65 or CBP/P300 interaction. As shown in Fig. 6a, Gm13568 was significantly interacted with NF-κB p65 and CBP/P300 in activated astrocytes by IL-9. Moreover, ChIP assay revealed that NF-κB p65, CBP/P300, RNA Pol II, and H3K27ac were differentially enriched in the Notch1 gene promoter in IL-9-activated astrocytes (Fig. 6b). Importantly, inhibition of Gm13568 significantly reduced NF-κB p65 and CBP/P300 enrichment in the Notch1 promoter (Fig. 6c). The data indicate that the interaction of Gm13568 with CBP/P300 may epigenetically regulate the transcription of the Notch1 gene.

### Notch1 knockdown in astrocytes represses inflammation and alleviates EAE in mice

**Fig. 7:**
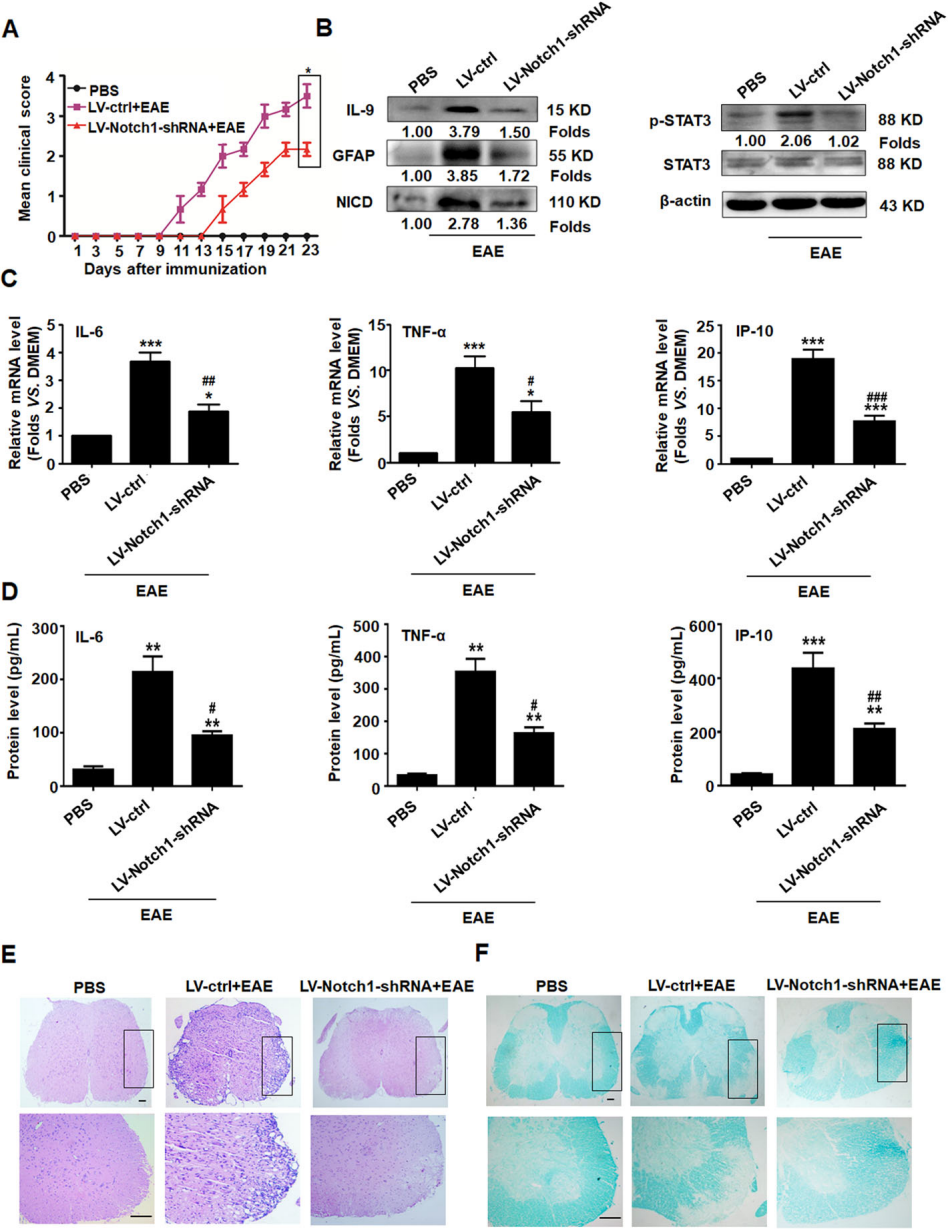
Knockdown of Notch1 in astrocytes suppresses inflammation and alleviates EAE in mice. Mice were subjected to recombinant lentiviruses, LV-ctrl or LV-Notch1-shRNA, for 7 days, followed by MOG_35-55_ immunization for 23 days (*n* = 10 mice per group). **a** The clinical scores of EAE mice with LV-ctrl and LV-Notch1-shRNA*.* **b** The expressions of IL-9, GFAP, NICD, and p-STAT3 in the spinal cords were detected using western blot assay. **c**, **d** The changes of IL-6, TNF-α, and IP-10 in the spinal cords and peripheral blood of the LV-ctrl and LV-Notch1-shRNA mice were evaluated by real-time PCR and CBA assay, respectively. ^*^p < 0.05, ^**^p < 0.01, ^***^p < 0.001 vs. PBS group; ^#^*p* < 0.05, ^##^*p* < 0.01, ^###^*p* < 0.001 versus LV-ctrl group (one-way ANOVA, the two-tailed Student’s *t* test). Results are represented as mean ± SEM. **e** The infiltration of inflammatory cells in spinal cords was investigated using hematoxylin and eosin (H&E) staining (scale bars, 50 μm). **f** The medullary sheath damages from spinal cords were observed via luxol fast blue (LFB) staining (scale bars, 50 μm). Boxed areas in the upper rows are presented enlarged underneath

To further clarify the role of Notch1 from astrocytes in EAE development, 1 × 10^7^ transforming units of recombinant lentivirus of LV-Notch1-shRNA specifically targeting astrocytes was injected into mice via the tail vein. After 7 days, EAE was induced by MOG _35-55_ for 23 days. The clinical score showed that silencing Notch1 in astrocytes not only delayed the onset of EAE but also relieved illness (Fig. 7a). Meanwhile, the production of IL-9 and the expression of GFAP were significantly decreased in EAE mice with LV-Notch1-shRNA (Fig. 7b). Furthermore, NICD and p-STAT3 protein expressions were also markedly downregulated in the spinal cords of the LV-Notch1-shRNA group (Fig. 7b). The levels of inflammatory cytokines such as IL-6, TNF-α and IP-10 were reduced in LV-Notch1-shRNA group (Fig. 7c, d). More importantly, H&E and LFB staining strongly displayed that LV-ctrl EAE mice had greater inflammatory cell infiltration and more severe demyelination lesions in the spinal cords. However, the EAE mice with LV-Notch1-shRNA had less inflammatory cell infiltration and slight demyelination lesions in the spinal cords (Fig. 7e, f). Collectively, these data reveal that Notch1 participants in the activation and hyperplasia of astrocytes, resulting in aggravation of inflammatory cell infiltration and spinal cords injury in EAE mice, which may be associated with an increased inflammatory reaction. On the contrary, knockdown of Notch1 reversed these results above.

### The inhibition of Gm13568 in astrocytes decreases the activation of the Notch1 pathway and ameliorates the EAE process in mice

**Fig. 8:**
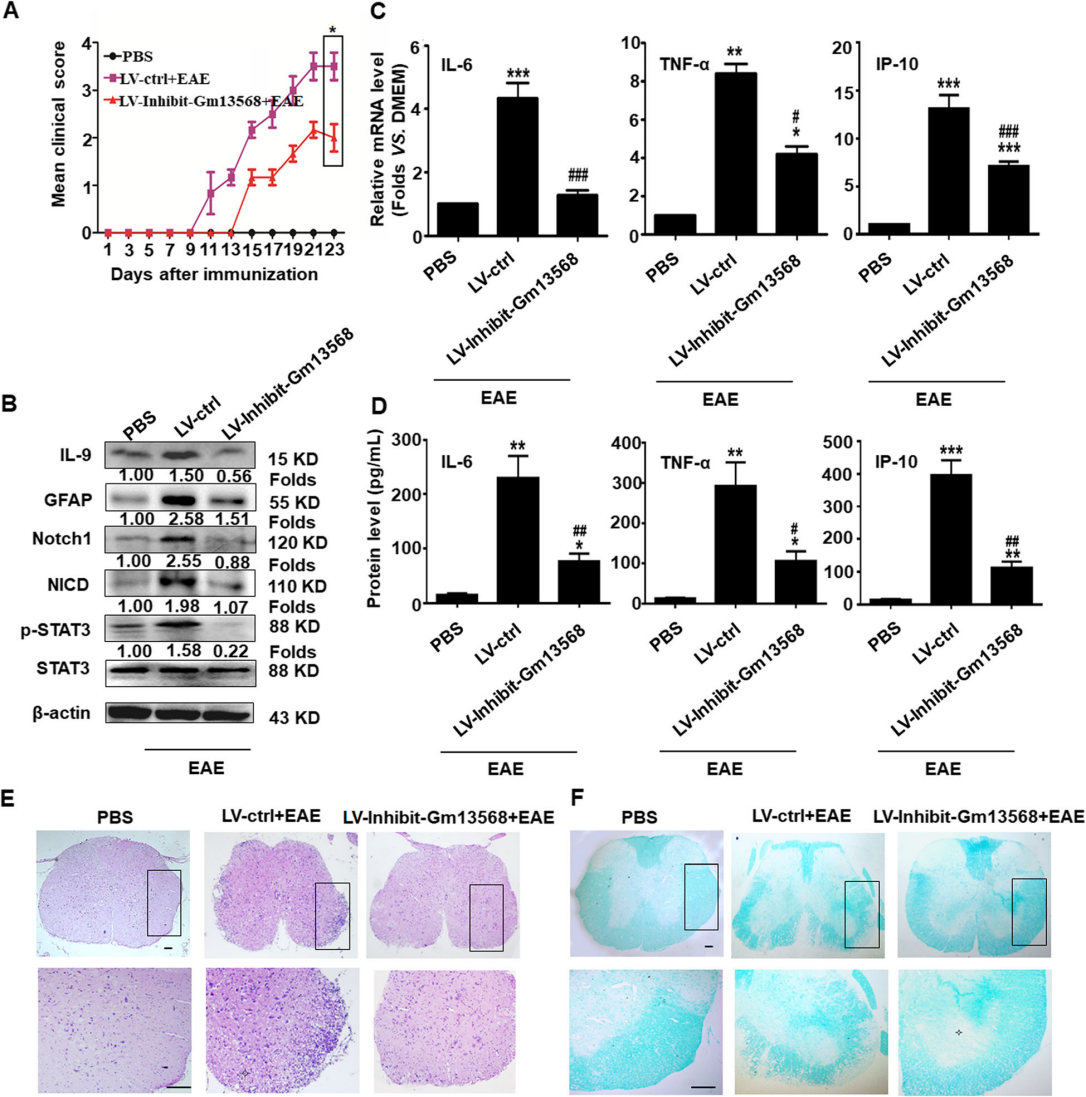
The inhibition of Gm13568 in astrocytes decreases Notch1 pathway activation and ameliorates the pathological process in EAE mice. On day 7 after administration with LV-ctrl or LV-Inhibit Gm13568, the mice were immunized with MOG_35-55_ for 23 days. **a** The clinical scores for EAE mice infected by LV-ctrl or LV-Inhibit Gm13568 (n = 10 mice per group). **b** The expression of IL-9 and GFAP and the activation of Notch1 pathway in the spinal cords were evaluated by Western blot assay. The results of a typical experiment are presented. **c**, **d** The levels of IL-6, TNF-α, and IP-10 from the spinal cords and peripheral blood in lentivirus-infected mice were analyzed by real-time PCR and CBA assay, respectively. ^*^p < 0.05, ^**^p < 0.01, ^***^p < 0.001 vs. PBS group; ^#^*p* < 0.05, ^##^*p* < 0.01, ^###^*p* < 0.001versus LV-ctrl group (one-way ANOVA, the two-tailed Student’s *t* test). **e** H&E staining for inflammatory cells infiltrations in spinal cords of mice (Scale bars, 50 μm). **f** LFB staining for the medullary sheath damage in spinal cords of mice (scale bars, 50 μm). Boxed areas in the upper rows are presented enlarged underneath

To verify whether Gm13568 regulating Notch1 in astrocytes contributes to the development of EAE, 1 × 10^7^ transforming units of recombinant lentivirus targeting astrocytes (LV-Inhibit-Gm13568 or LV-ctrl) was administrated to mice via the tail vein, and then EAE was induced after 7 days. As shown in Fig. 8a, inhibiting Gm13568 in astrocytes slowed down the EAE development. At the same time, the production of IL-9 was significantly reduced compared to the LV-ctrl mice (Fig. 8b). The expressions of GFAP, Notch1, NICD, and p-STAT3 were dramatically decreased in the LV-Inhibit-Gm13568 mice (Fig. 8b). Similarly, the production of IL-6, TNF-α, and IP-10 was also markedly diminished in LV-Inhibit-Gm13568 group (Fig. 8c, d). In addition, H&E and LFB staining showed that inflammatory cell infiltration and demyelination lesions in white matter were obviously reduced in LV-Inhibit-Gm13568 group, compared to the LV-ctrl mice (Fig. 8e, f). Taken together, these data imply that the lncRNA Gm13568 regulates the Notch1 pathway in astrocytes, thus controlling reactive astrocytosis, inflammatory cytokine secretion and spinal cord injury in mice, in turn affecting the EAE process.

## Discussion

**Fig. 9:**
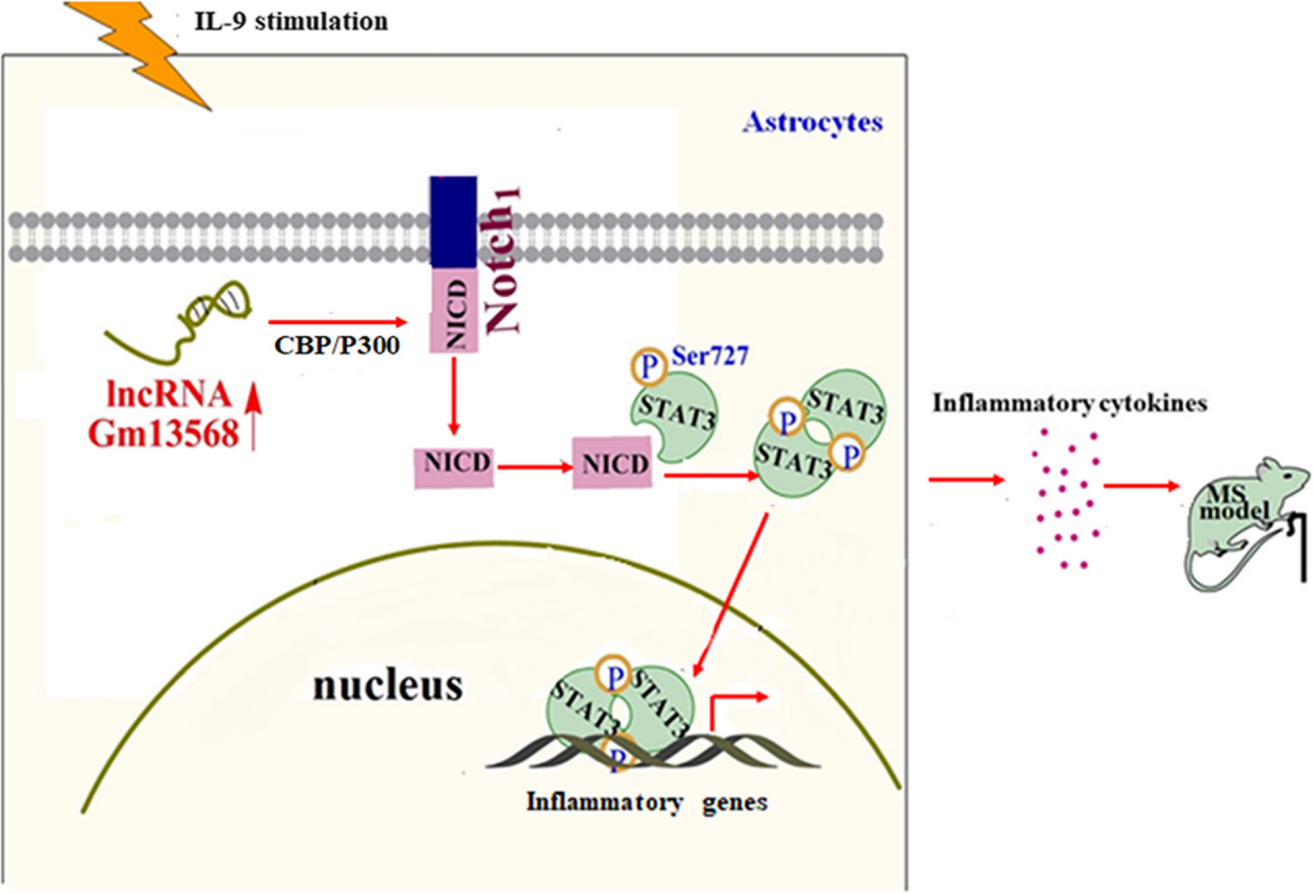
Schematic representation of lncRNA Gm13568 contributing to the pathogenesis of EAE mice via regulation of the Notch1 pathway. Under IL-9 stimulation, Gm13568 is upregulated in astrocytes, which promotes the expression and activation of Notch1 via interacting with CBP/P300, in turn contributing to the release of inflammatory cytokines, thus aggravating the EAE development

Astrocytes play important roles in MS by recruiting lymphocytes, confining inflammation and contributing to tissue damage [31, 32]. It has also been reported that lncRNAs have significant impacts on normal neural development and the progression of neurodegenerative diseases [17]. Our previous studies have demonstrated that lncRNAs differentially expressed in the activated astrocytes are closely related to EAE process [19]. Here, we confirm that inhibiting lncRNA Gm13568, an upregulated lncRNA both in IL-9-activated astrocytes and in EAE mice, reduces inflammatory cytokine production in astrocytes and delays the EAE process through decreasing Notch1 expression and signal activation by interaction with NF-κB p65 and CBP/P300 (Fig. 9).

In CNS autoimmunity, reactive astrocytes can contribute to a detrimental positive-feedback loop of inflammation, which accelerates inflammatory cascades and aggravates neurological disorders [3]. Response to inflammatory stress, many genes relevant to inflammation, oxidative stress, immune receptors, and BBB disruption are increased in astrocytes [33]. It has been shown that astrocytes aggravate BBB disruption, increase chemokines production, and facilitate T cell migration to the CNS upon IL-9 stimulation [8, 9]. Our data showed that mRNA transcription and protein expression of IL-9 in the spinal cords of EAE mice were gradually increased along with the severity of EAE. Notably, the expression of GFAP and the secretion of pro-inflammatory cytokines were also significantly upregulated along with the severity of EAE, which implied the activation and hyperplasia of reactive astrocytes. However, the understanding of the role of IL-9 in promoting astrogliosis is still very limited.

The Notch pathway can modulate immune response and inflammatory processes, which are clinically critical in MS. Emerging evidence indicates the Notch signal regulates the development and maturation of microglia, oligodendrocytes, and CD4^+^T cells in MS lesions, which are associated with MS pathology [34]. Inhibiting the Notch1 pathway in oligodendrocytes improved remyelination in EAE mice and in the cuprizone (CPZ) demyelination model [34, 35]. It has been demonstrated that Notch-1 signaling is a critical regulator of intracerebral hemorrhage (ICH)-induced reactive astrogliosis [15]. Our results showed that the expression of Notch1, NICD, and p-STAT3 was consistent with that of GFAP and inflammation cytokines production in EAE and in reactive astrocytes with IL-9 stimulation. Importantly, knockdown Notch1 in astrocytes not only decreased inflammatory production both in vivo and in vitro, but also reduced inflammatory cell infiltration and demyelination lesion in the spinal cords of EAE mice. Yet, the underlying mechanisms behind Notch1 modulation in astrocytes are far from clear in MS.

In recent years, lncRNAs having been shown to play vital roles in diverse biological processes ranging from development to immune responses. In the CNS, they diversely participate in neuroinflammation, synapse plasticity, synaptogenesis, and memory formation [30, 36, 37]. Accumulating evidence has indicated that lncRNAs are key regulatory molecules involved in MS pathogenesis [38–42], but these data mainly focused on the regulation of Th cells and microglia by lncRNAs. Given that astrocytes play essential roles in MS process, it is crucial to understand the role of lncRNAs in astrocytes in MS. To date, the functional characterization of lncRNAs during astrocyte activation and MS process has not been fully uncovered. Our previous study identified the changes of lncRNA expression profiles both in EAE mice and in activated astrocytes with IL-9 stimulation [19]. We found that the sequence of co-upregulated lncRNA Gm13568 in vivo and in vitro were complementary to the 9075-9497 locus of the sense chain in the Notch1 gene, and the time course of Gm13568 changes was consistent with that of Notch 1 in IL-9-stimulated astrocytes. lncRNAs are known to control the expression of genes as well as contribute to the pathogeneses of diseases. Therefore, we propose that Gm13568 may positively regulate the expression of Notch1 gene in astrocytes, therefore contributing to the EAE pathogeneses. In this study, our data supports this hypothesis. Our study revealed that the inhibition of Gm13568 decreased Notch1 expression and pathway activation in astrocytes, as well as attenuated reactive astrocytosis both in vitro and in vivo, which elicited the reduction of inflammatory cytokines and chemokines production, thus alleviating inflammatory cell infiltration and demyelination lesion in EAE mice. However, it is still not very clear about the mechanisms by which lncRNAs act on Notch1in astrocytes.

It has been demonstrated that lncRNAs can modulate protein-coding gene expression in chromatin remodeling and histone modification, which are always associated with the CBP/p300 complex, the CoREST/REST complex and the Polycomb Repressive Complex 2 (PRC2) complex [16, 36]. In the present study, RIP and ChIP assay showed that Gm13568 associated with NF-κB p65 and CBP/P300 was enriched in the promoter of Notch1gene. These data suggest that the interaction of Gm13568 and CBP/P300 might epigenetically regulate Notch1 expression in activated astrocytes and EAE mice.

## Conclusion

In summary, our present evidence reveals that lncRNA Gm13568 induced by IL-9 in astrocytes regulates Notch1 expression through the interaction with CBP/P300 and then promotes Notch1 pathway activation, which is involved in the production of inflammatory cytokines and chemokines in astrocytes, in turn affecting EAE development. Thus, these findings emphasize that the lncRNA derives from astrocytes may play key roles in neuroinflammation and pathogenesis of MS.

## Supplementary Information

**Additional file 1: Fig. S1** The neurons and astrocytes were infected by lentivirus with GFAP promoter. The HT-22 cell strain of mouse hippocampal neuron and primary mouse astrocytes were infected by the lentivirus with the astrocyte-specific promoter of GFAP or GFP (without GFAP promoter). At 72 h after lentivirus infection, GFAP or GFP expression were observed under fluorescence scope (Scale bars, 50 μm).

**Additional file 2: Table S1.** The list of primer sequences for qPCR assay.

## Data Availability

All data generated or analyzed during this study are included in this published article and its supplementary information files.

## Abbreviations

IL-9: Interleukin-9
CBP/P300: CREB-binding protein (CBP) and p300
MS: Multiple sclerosis
EAE: Experimental autoimmune encephalomyelitis
STAT3: Signal transducer and activator of transcription 3
IL-6: Interleukin-6
TNF-α: Tumor necrosis factor-α
IP-10: Interferon-gamma induced protein 10
MCP-1: Monocyte chemoattractant protein-1
CNS: The central nervous system
GFAP: Glial fibrillary acidic protein
NICD: Notch1 intracellular domain
MOG: Myelin oligodendrocyte glycoprotein
CBA: Cytometric Bead Array
RIP: RNA immunoprecipitation
ChIP: Chromatin immunoprecipitation
