# Supplementary figures and images for: IL-9-triggered lncRNA Gm13568 regulates Notch1 in astrocytes through interaction with CBP/P300: contribute to the pathogenesis of experimental autoimmune encephalomyelitis
Source: J Neuroinflammation. 2021 May 11;18:108. doi: 10.1186/s12974-021-02156-5 (PMC8112022; doi:10.1186/s12974-021-02156-5)

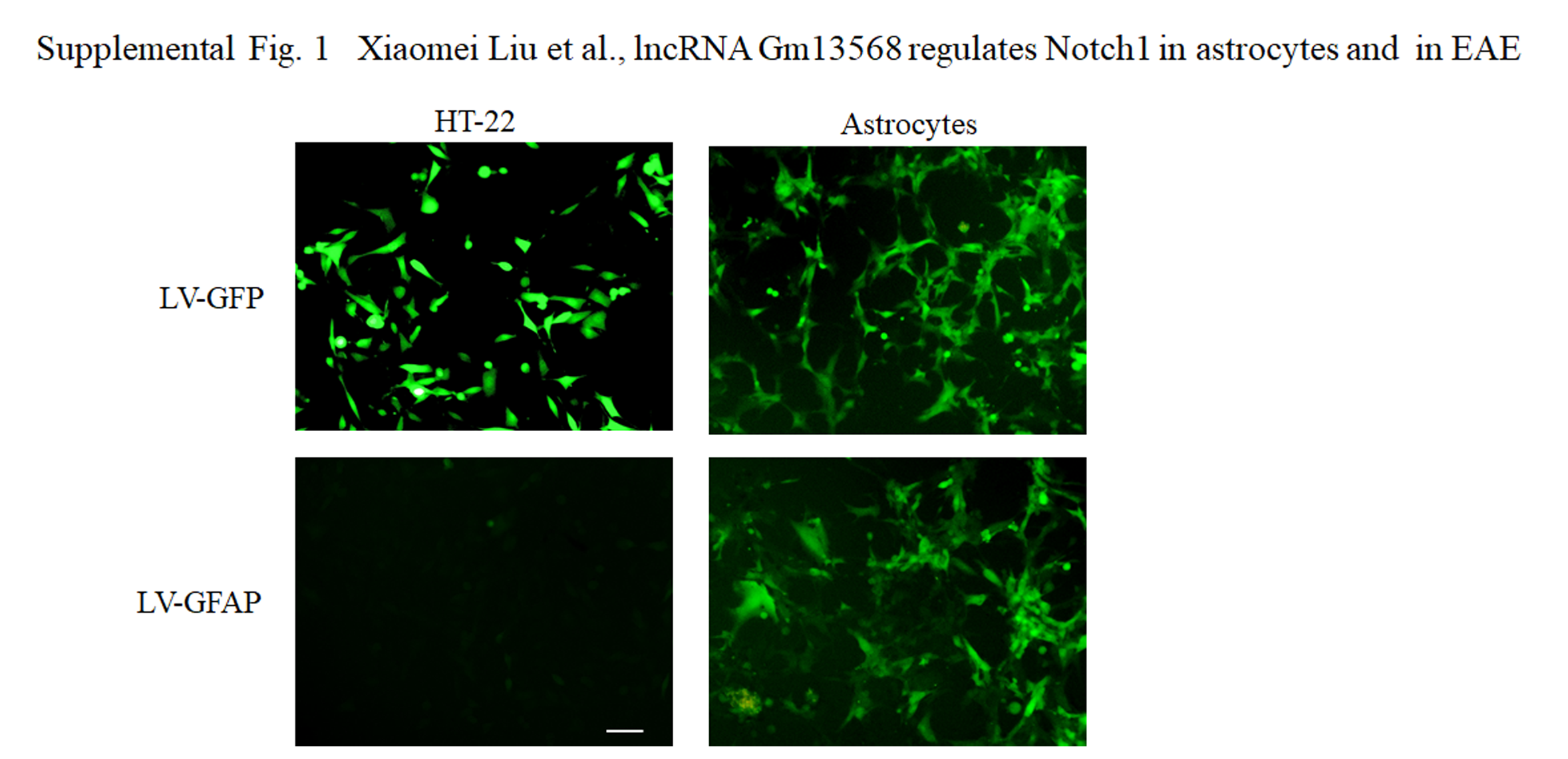

Supplement: Supplementary file 1 — Additional file 1: Fig. S1 The neurons and astrocytes were infected by lentivirus with GFAP promoter. The HT-22 cell strain of mouse hippocampal neuron and primary mouse astrocytes were infected by the lentivirus with the astrocyte-specific promoter of GFAP or GFP (without GFAP promoter). At 72 h after lentivirus infection, GFAP or GFP expression were observed under fluorescence scope (Scale bars, 50 μm). [file 12974_2021_2156_MOESM1_ESM.tif]
