# Supplementary material for: IL-9-triggered lncRNA Gm13568 regulates Notch1 in astrocytes through interaction with CBP/P300: contribute to the pathogenesis of experimental autoimmune encephalomyelitis
Source: J Neuroinflammation. 2021 May 11;18:108. doi: 10.1186/s12974-021-02156-5 (PMC8112022; doi:10.1186/s12974-021-02156-5)
Supplement: Supplementary file 2 — Additional file 2: Table S1. The list of primer sequences for qPCR assay. [file 12974_2021_2156_MOESM2_ESM.doc]

**Xiaomei Liu et al., lncRNA Gm13568 regulates Notch1 in astrocytes and in EAE**

**Supplementary Table 1. Primer sequences for real-time PCR**

| **Primer Name** | **Sequence (5’ to 3’)** |
| --- | --- |
| **Primers for mRNAs** |  |
| IL-9 | Fw TTTTGCTCTTCAGTTCTGTGCTG |
|  | Rev CGGTGTGGTACAATCATCAGTTG |
| IL-6 | Fw CCACGGCCTTCCCTAC |
|  | Rev AAGTGCATCATCGTTGT |
| TNF-α | Fw CCACCACGCTCTTCTGTCTACTG |
|  | Rev GCCATAGAACTGATGAGAGG |
| IP-10 | Fw CGTCATTTTCTGCCTCATCCT |
|  | Rev TGGTCTTAGATTCCGGATTCAG |
| MCP-1 | Fw TTAAAAACCTGGATCGGAACCAA |
|  | Rev GCATTAGCTTCAGATTTACGGGT |
| Notch1 | Fw ATGGAGGATGGCAGTGATGT |
|  | Rev ATGGAGGATGGCAGTGATGT |
| Actin | Fw CGTGGGCCGCCCTAGGCACCA |
|  | Rev TTGGCCTTAGGGTTCAGGGGGG |
|  |  |
| **Primers for lncRNA** |  |
| Gm13568 | Fw TAGCAAAATGTGGACAACCATC |
|  | Rev GCCTAATAGATATGACCAGACGC |
|  |  |
| **Primers for RIP** |  |
| Gm13568-1 | Fw TAGGCATGGCACAGACACTG |
|  | Rev AAGAGCACAACCCAGGATGG |
| **Primers for ChIP** |  |
| p65-1 | Fw TTTCCCAGGCCACCTAAGTC |
|  | Rev CCGAGTCTAGACGTTCCACG |
| p65-2 | Fw CACCTAAGTCGGACACAGCC |
|  | Rev GTGCCTTGCTGGACAGATTG |
| p65-3 | Fw CCACCTAAGTCGGACACAGC |
|  | Rev GATTGCGCTCTCGATTCCCA |
| p300-1 | Fw TCTAGACTCGGGTTCCTGCT |
|  | Rev TGACTTCCGGAACGCTTGAC |
| p300-2 | Fw GTGGAACGTCTAGACTCGGG |
|  | Rev TTGAGGCCAGGCACTCTTAG |
| p300-3 | Fw CTGGGAATCGAGAGCGCAAT |
|  | Rev GTAGAAGGGAGTTCGGGGCT |
| H3k27ac-1 | Fw GGAATCGAGAGCGCAATCTG |
|  | Rev GAGGCCAGGCACTCTTAGC |
| H3k27ac-2 | Fw GCACTAGTCCTTGGGGAGTT |
|  | Rev GATTGAGGCCAGGCACTCTTA |
| H3k27ac-3 | Fw TGAGAAGGGTGACCGTGGAA |
|  | Rev GCCCGCAGCCTCTGTAGAA |
| Poly II RNA-1 | Fw GGAGGGACAGGGTCCACA |
|  | Rev GGCAGAGGCACTAGTGAGG |
| Poly II RNA-2 | Fw GCCTCACTAGTGCCTCTGC |
|  | Rev CCGGGCTCGTTCCTTCAC |
| Poly II RNA-3 | Fw TCCTTAGATCCTGGCTCGGG |
|  | Rev TGTGGACCCTGTCCCTCC |
